# State Boredom Partially Accounts for Gender Differences in Novel Lexicon Learning

**DOI:** 10.3389/fpsyg.2022.807558

**Published:** 2022-08-29

**Authors:** Hua Wang, Yong Xu, Hongwen Song, Tianxin Mao, Yan Huang, Sihua Xu, Xiaochu Zhang, Hengyi Rao

**Affiliations:** ^1^Center for Magnetic Resonance Imaging Research & Key Laboratory of Applied Brain and Cognitive Sciences, School of Business and Management, Shanghai International Studies University, Shanghai, China; ^2^Institute of Linguistics, Shanghai International Studies University, Shanghai, China; ^3^Department of Radiology, the First Affiliated Hospital of USTC, Hefei National Laboratory for Physical Sciences at the Microscale and School of Life Science, Division of Life Science and Medicine, University of Science & Technology of China, Hefei, China; ^4^Academy of Psychology and Behavior, Tianjin Normal University, Tianjin, China

**Keywords:** gender, state boredom, lexicon learning, mediation analysis, trait boredom

## Abstract

Gender plays an important role in various aspects of second language acquisition, including lexicon learning. Many studies have suggested that compared to males, females are less likely to experience boredom, one of the frequently experienced deactivating negative emotions that may impair language learning. However, the contribution of boredom to gender-related differences in lexicon learning remains unclear. To address this question, here we conducted two experiments with a large sample of over 1,000 college students to explore the relationships between gender differences in boredom and lexicon learning. In Experiment 1, a cohort of 527 participants (238 males) completed the trait and state boredom scales as well as a novel lexicon learning task without awareness of the testing process. In Experiment 2, an independent cohort of 506 participants (228 males) completed the same novel lexicon learning task with prior knowledge of the testing procedure. Results from both experiments consistently showed significant differences between female and male participants in the rate of forgetting words and the state boredom scores, with female participants performing better than male participants. Furthermore, differences in state boredom scores partially explained differences in the rate of forgetting words between female and male participants. These findings demonstrate a novel contribution of state boredom to gender differences in lexicon learning, which provides new insights into better language-learning ability in females.

## Introduction

Billions of students are learning second (L2) or foreign languages (FLL) every year in the globalized contemporary world. Gender-related differences have been consistently observed in various aspects of language learning. For example, previous research has demonstrated that female learners are likely to perform better than male learners in multiple language learning, such as clearer pronunciation, politer language, better oral communication, and faster vocabulary learning speed (e.g., Gass and Varonis, 1986; Lynn et al., 2005; Van et al., 2015; Ng, 2018; Syafrizal and Putri, 2020).

It is well-known that the success of L2 or FLL depends on learners’ emotional status (Krashen, 1981), which includes affective, cognitive, motivational, and peripheral physiological processes. Boredom is one of the most experienced emotions during learning and education (Pekrun, 2006; Goetz and Hall, 2014; Putwain et al., 2018; Li, 2021). According to the Control-Value Theory (CVT), boredom is a deactivating negative emotion resulting from an activity that lacks incentive value and perceived controllability or high control/low-demands conditions implying no sufficient challenge that reduces the incentive value of the activity (Pekrun, 2006). More recently, the Meaning and Attentional Components (MAC) model posits that boredom may result from mismatches between cognitive demands and available mental resources, or mismatches between activities and valued goals (Westgate and Wilson, 2018; Westgate, 2020). Moreover, boredom could hinder academic improvement by affecting perceived meaning (Eastwood et al., 2012; Tam et al., 2021) or disrupting the attention control system in the learners (Suárez-Pellicioni et al., 2016). There is also evidence suggesting that males are more likely to feel bored than females (Watt and Ewing, 1996; Watt and Vodanovich, 1999; Liu et al., 2013). However, scant attention has been paid to the prevalent emotional status of boredom in the L2 and FLL context (Kruk, 2019; Li et al., 2020; Li, 2021), and the same is true for gender differences when it comes to boredom. To date, the contributions of boredom to gender differences in lexicon learning remain unknown.

To address this question, here we conducted two experiments to explore the relationships between gender differences in boredom and lexicon learning in a large sample of over 1,000 college students. In Experiment 1, a cohort of 527 students completed the trait and state boredom scales as well as a lexicon learning task without awareness of the testing process. In Experiment 2, an independent cohort of 506 students completed the same lexicon learning task with prior knowledge of the testing procedure. We expected to observe significant gender differences between male and female participants in the boredom scores, which would contribute to their differences in lexicon learning performance.

## Literature Review

### Gender Differences in Lexicon Learning

A number of previous studies on gender differences in lexicon learning have shown that gender is a critical variable that influences vocabulary learning performance. Some researchers reported that male students were superior in understanding and using vocabulary (Gass and Varonis, 1986; Lynn et al., 2005). In contrast, others highlighted that compared to male students, female students performed better in vocabulary memorization (Sunderland, 2000; Scheiber et al., 2015), pronunciation (Syafrizal and Putri, 2020), acquisition size, and general proficiency (Gu, 2002). Concerning the semantic fields, female students were better at acquiring vocabulary describing story characters, whereas male students were better at acquiring vocabulary related to sports and geography (Jiménez, 2010). On vocabulary learning strategies, male students tended to use form-focused memory, cognitive processes, and metacognitive monitoring more frequently, while female students possessed a disposition to adopt meaning-focused cognitive strategies and metacognitive planning strategies more frequently (Van et al., 2015; Ng, 2018). In summary, findings on gender differences depend on the aspects examined, and little research has been conducted to examine gender effects on novel lexicon learning achievement.

The observed variability may be explained by the following reasons. The Gender Role Theory posits that prevalent gender stereotypes are culturally shared expectations for gender appropriate behaviors. Females and males acquire appropriate behaviors and attitudes from the sociocultural environment they grow up in (Eagly and Karau, 2002; Bryła-Cruz, 2021). The biological viewpoint suggests that gender difference also depends on cognitive ability and learning style, which are derived from fundamental physiological differences, such as those in the development of the brain or higher-level cortical functions (de Lima Xavier et al., 2019). Regardless of primarily cultural or biological factors, previous educational studies have proven that gender difference manifestly influences students’ academic achievements (Główka, 2014).

Considering previous studies, results regarding gender differences in the lexical acquisition are inconclusive. Moreover, most studies have concentrated on the gender differences in pre-university education (e.g., Chee et al., 2005; Aldosari et al., 2017) differences in novel lexicon learning achievement among university students may contribute to our understanding of the whole phenomenon of gender differences in L2 or FLL. The gender gap in favor of L2 or FLL female learners also requires further research in multiple aspects of language competence, including novel lexicon learning.

### Gender Differences in Boredom

Boredom can be defined as a dissatisfying state of wanting, but being unable, to engage in the desirable activity (Eastwood et al., 2012). The attention mismatch hypothesis proposes that boredom may occur when there is a mismatch between task requirements and attention ability (Gerritsen et al., 2014). Boredom could be further divided into two subtypes: trait boredom and state boredom (Farmer and Sundberg, 1986). Trait boredom consists of external stimuli and internal stimuli (Vodanovich et al., 2005). An early study of boredom posited that people with increased susceptibility to boredom are less psychosocially developed and thereby have reduced psychosocial abilities to deal with various situations in life (Watt and Vodanovich, 1999). Furthermore, individuals with a high trait of boredom tend to struggle with attention in daily life (Malkovsky et al., 2012) and are more vulnerable to mood disorders like depression (Goldberg et al., 2011). As a chronic tendency to be bored, trait boredom or boredom proneness is also related to various mental health and behavior problems, such as drug use disorder (LePera, 2011), low life meaning (Fahlman et al., 2009) and impulsivity disorders (Malkovsky et al., 2012).

In contrast to trait boredom, state boredom reflects more transient reactions to specific situations, including inattention, time perception, low arousal, high arousal, and disengagement (Liu et al., 2013). State boredom is typically associated with perceptions of time passing by slowly and failures of attention (Pekrun et al., 2010; Eastwood et al., 2012; Hunter and Eastwood, 2016; Westgate, 2020). The perception of meaninglessness or task unimportance is an independent determinant of state boredom (Fahlman et al., 2009; Anusic et al., 2016; Van Tilburg and Igou, 2017b; Chan et al., 2018; Westgate and Wilson, 2018). State boredom may affect individual preference and behavior through stimulation seeking (Van Tilburg and Igou, 2012), awakening curiosity about the environment (Lomas, 2017), or reflecting the self-regulation function of state boredom (Miao and Xie, 2019). Individuals with a high-level state of boredom have been associated with increased hostility (Van Tilburg and Igou, 2012), riskier decisions (Matthies et al., 2012), and poor sustained attention (Westgate, 2020). Taken together, trait boredom and state boredom may reflect different dimensions of boredom and have different effects on language learning.

Most previous studies reported gender-related differences in boredom with males showing greater boredom than females, which may be attributed to differences in personality (Liu et al., 2013) or susceptibility to being bored (Vodanovich and Kass, 1990). For example, compared with females, males are more extroverted, lively and active, and easily bored of learning activities, as they prefer to pursue novel stimulation (Liu et al., 2013). It was reported that female students might experience less boredom due to lacking the ability to perceive interest and significance from the environment (Watt and Vodanovich, 1999), while male students had higher levels of boredom and greater boredom proneness than female students on external stimulation (Von Gemmingen et al., 2003; Vodanovich et al., 2011). Concerning state boredom, previous studies reported that male students yielded significantly higher scores on the state boredom scale (Liu et al., 2013) and different time perceptions than female students (Pawlak et al., 2020).

However, null or even reversed findings on gender differences in boredom have also been reported. For example, McLeod and Vodanovich (1991) and Watt and Ewing (1996) reported no differences between males and females in boredom proneness. Seib and Vodanovich (1998) even reported that males were less likely to experience boredom than females, which may have been due to their inability to self-generate participation. One possible explanation for these discrepant findings is that boredom is multifaceted, and that gender differences may be more pronounced in one subtype of boredom but not the other. Another possible explanation is that gender differences in boredom may not be fully manifested until people reach a particular age level. Nevertheless, more research is necessary to further clarify gender differences in state and trait boredom in large samples and repeatable studies.

### Relationships Between Boredom and Learning Performance

It is well-known that learners’ emotional status plays an important role in academic performance. As one type of frequently experienced deactivating negative emotional status, boredom is likely to impair learning and academic performance (Pekrun, 2006; Putwain et al., 2018; Kruk and Zawodniak, 2020). The Affective Filtering Hypothesis (Krashen, 1981) posits that language input must pass through an emotional filter before it can be absorbed, and that the stronger the filter, the more language input is suppressed in the brain, leading to poorer achievements in language learning. Numerous empirical studies have reported the negative effects of boredom on academic performance. For example, Frenzel et al. (2007) reported that fifth to tenth graders’ boredom levels during math classes correlated negatively with their math achievement. Pekrun et al. (2010) found that undergraduate students’ boredom negatively predicted their end-of-year performance. Using a longitudinal design, Ahmed et al. (2013) reported that change in seventh graders’ boredom over one school year was negatively associated with math achievement. However, an early study reported small but positive correlations between fifth to ninth graders’ boredom and grade point average and test scores (Larson and Richards, 1991), suggesting that the relationships between boredom and academic performance may not always be negative.

Although previous literature has demonstrated gender-related differences in boredom (e.g., Vodanovich and Kass, 1990; Von Gemmingen et al., 2003; Vodanovich et al., 2011; Liu et al., 2013; Pawlak et al., 2020) as well as in language learning (e.g., Sunderland, 2000; Gu, 2002; Lynn et al., 2005; Jiménez, 2010; Scheiber et al., 2015; Ng, 2018), whether gender differences would be similar in subtypes of boredom (i.e., state boredom or trait boredom) remains unclear. Moreover, few if any studies have differentiated the effects of trait boredom and state boredom on language learning and examined the contributions of these boredom subtypes to gender differences in lexicon learning. To address this knowledge gap, the Multidimensional State Boredom Scale (MSBS; Liu et al., 2013) and the Trait Boredom Scale (TBS; Huang et al., 2010) were applied to measure state and trait boredom levels, respectively, in a large sample of college students before they completing a novel lexicon learning task and the tests. Similar to the findings from previous studies, we expected that females would experience less state and trait boredom during the lexicon learning. We also wanted to examine whether state or trait boredom would be a mediator variable for the lexicon learning ability difference between female and male students.

## Methodology

### Participants

We recruited a total of 1,070 non-language major students from a college for this study, including 550 participants for Experiment 1 and 520 participants for Experiment 2. Twenty-three participants (4.18%) were excluded from Experiment 1 and fourteen participants (2.69%) were excluded from Experiment 2 due to incompetence or failure to complete the whole study. Data from 1,033 participants were included in the final data analysis, including 527 participants (238 male; mean age = 19.73 ± 2.02 years) for Experiment 1, and 506 participants (228 male; mean age = 19.80 ± 1.45 years) for Experiment 2. All participants reported no history of psychological and psychiatric disorders. There were no differences between male and female participants in age or years of education in both Experiment 1 and Experiment 2 (all *p* > 0.1). The study protocol was approved by the Ethics Committee of Shanghai International Studies University. Participants provided written informed consent before the experiment and received monetary rewards for their participation.

### Measures and Materials

The Multidimensional State Boredom Scale (MSBS; Liu et al., 2013) and the Trait Boredom Scale (TBS; Huang et al., 2010) was adopted to assess the participants’ levels of state boredom and trait boredom. The MSBS scale includes 24 items divided into five dimensions: (1) *Inattention* refers to having difficulty focusing attention on the current environment or activity. A higher score on this dimension, the harder it is for individuals to concentrate. (2) *Time perception* refers to the excessively slow perception of time. A higher score on this dimension, the more slowly they feel that time passes. (3) *Low arousal* refers to feelings of calmness and depression. This is also a manifestation of negative experiences in the state of boredom. To a certain extent, high state boredom can be reflected by negative emotions. (4) *High arousal* refers to feelings of energy for pleasurable states (e.g., excitement), or tension for unpleasant states (e.g., fear). A higher score on this dimension indicates a higher level of uncontrollable restlessness. (5) *Disengagement* is a lack of participation in current activities and desire to participate in more exciting activities. This emotion could affect people’s concentration on their current tasks. All items on the scales are scored from “1 = not agree at all” to “7 = completely agree.” A higher total score on the MSBS represents a higher level in the state of boredom. The MSBS scale has a Cronbach’s alpha of 0.83 in the present sample, suggesting good internal consistency in the study.

The TBS scale includes 30 items divided into two dimensions: external stimuli and internal stimuli. The former dimension includes four factors: monotony, loneliness, tension, and restraint. The latter dimension consists of two elements: self-control and creativity (Vodanovich et al., 2005). These items are all scored from “1 = not at all” to “5 = completely true.” In the current study, we used the total score to measure the individual’s boredom proneness. A higher total score indicates a higher level of trait boredom. The TBS scale has a Cronbach’s alpha of 0.79 in the present sample, also suggesting good internal consistency in the study.

### Pseudoword-Chinese List

The pseudoword-Chinese list was used to measure the result of lexicon learning. The list includes 16 pseudowords, which are coined according to real words and their number of syllables. There are two criteria when selecting pseudowords: (1) eliminating the pseudowords that may lead to the association of real foreign words at a sound or morphological level; (2) using monosyllables, disyllables, trisyllables, and keeping the number of vowels and consonants approximately equal (Gathercole et al., 1991). Each pseudoword is matched with a neutral Chinese meaning. The pseudoword-Chinese list is as follows (see Table 1).

**Table 1:** Pseudoword-Chinese list.

Pseudo-word	Chinese	Pseudo-word	Chinese	Pseudo-word	Chinese	Pseudo-word	Chinese
thicult	时间	bidt	坚硬	hond	早	jis	硬件
viulu	下午	deppelate	大的	glitow	飞船	bannow	孩子
blonter	变成	tuwhep	道路	soku	商店	bomme	储存
mef	经历	prindle	告诉	ganner	工作	glisterin	明白

### Procedure

**Figure 1:**
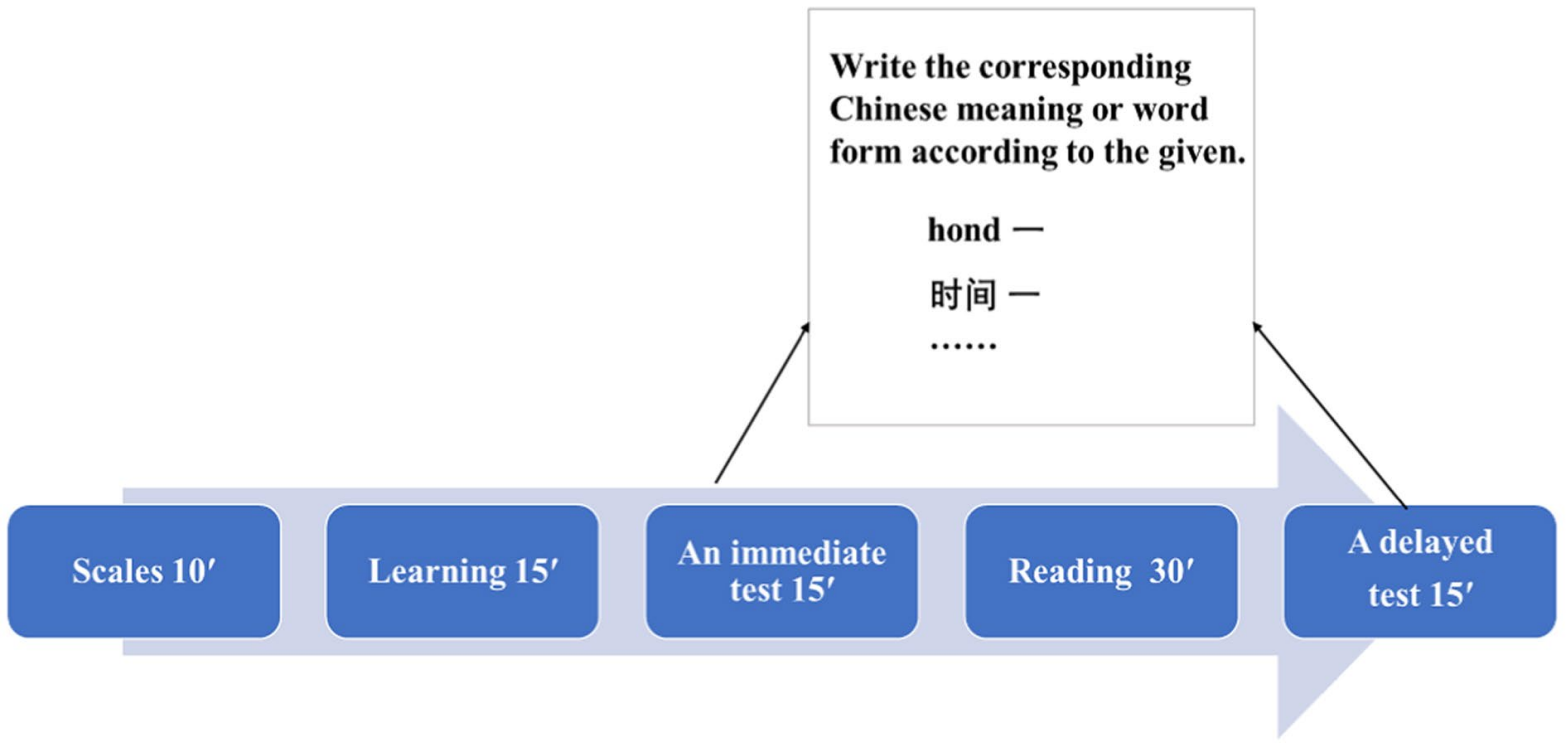
Diagram of the experiment process and the test task.

We first conduct Experiment 1 to explore whether trait boredom, state boredom, or both had a significant effect on novel lexicon learning. Then, we conducted Experiment 2 to replicate the main findings in Experiment 1. To measure the level of boredom of the participants and the effects of novel lexicon learning in Experiment 1 and Experiment 2, we adopted the following experiment process (see Figure 1). First, participants’ boredom experience was measured with the corresponding boredom scales. After finishing the boredom scales, the pseudoword-Chinese pairs were learned for 15 min, and immediate testing was carried out for about 10 min. Then, participants were arranged to have a 30 min reading. Finally, participants completed a delayed cued recall test in which they were required to write the corresponding Chinese meanings or pseudowords according to the given pseudo-words. The purpose of performing a delayed test as a retest was to measure the relatively stable learning effect (Ke and Dong, 2001). There was a total score of 16 points as one point was given for each correct answer.

At the beginning of learning and tests, participants were asked “How bored are you right now?” with a corresponding Likert scale ranging from 0 (*not at all*) to 8 (*extremely*) presented on the top of the pseudoword list and each test paper. One-way ANOVA analyses were used to confirm that the level of state boredom did not change significantly over the whole process. To rule out the potential confounding factor that the novel lexicon learning task itself may induce boredom, we excluded those participants who reported significant differences in state boredom between the two conditions in the data analyses.

### Statistical Analysis

All data were processed and analyzed by using the statistical software SPSS 22.0. Since the difference between the immediate score (the number of correct words) and the delayed score was not always inversely proportional to the learning effect in our experiments, we calculated the individual word forgetting rate as the effect of novel lexicon learning: Forgetting Rate (FR) = (score_immediate_−score_delayed_)/(score_immediate_). Higher FRs reflected poorer learning effects. Independent sample t-tests were used for comparing the differences in the studied variables between males and females. Pearson correlation analyses were used to examine the correlations between boredom scores and FRs. A hierarchical multiple regression analysis was conducted to further estimate the effect of gender, state and trait boredom as predictors of FRs. The PROCESS 3.3 program and bootstrap method were employed to verify the mediating effects of boredom.

Based on the literature review, the following four hypotheses were tested in this study: (1) female participants would show better performance (lower FRs) than male participants in the tests after the lexicon learning task; (2) female participants would show lower state and trait boredom than male participants before the learning task; (3) greater state and trait boredom level would be associated with a worse outcome of the lexicon learning task; and (4) state or trait boredom may be a mediator variable for the relationships between gender and lexicon learning.

## Results

### Gender and Learning Performance

**Figure 2:**
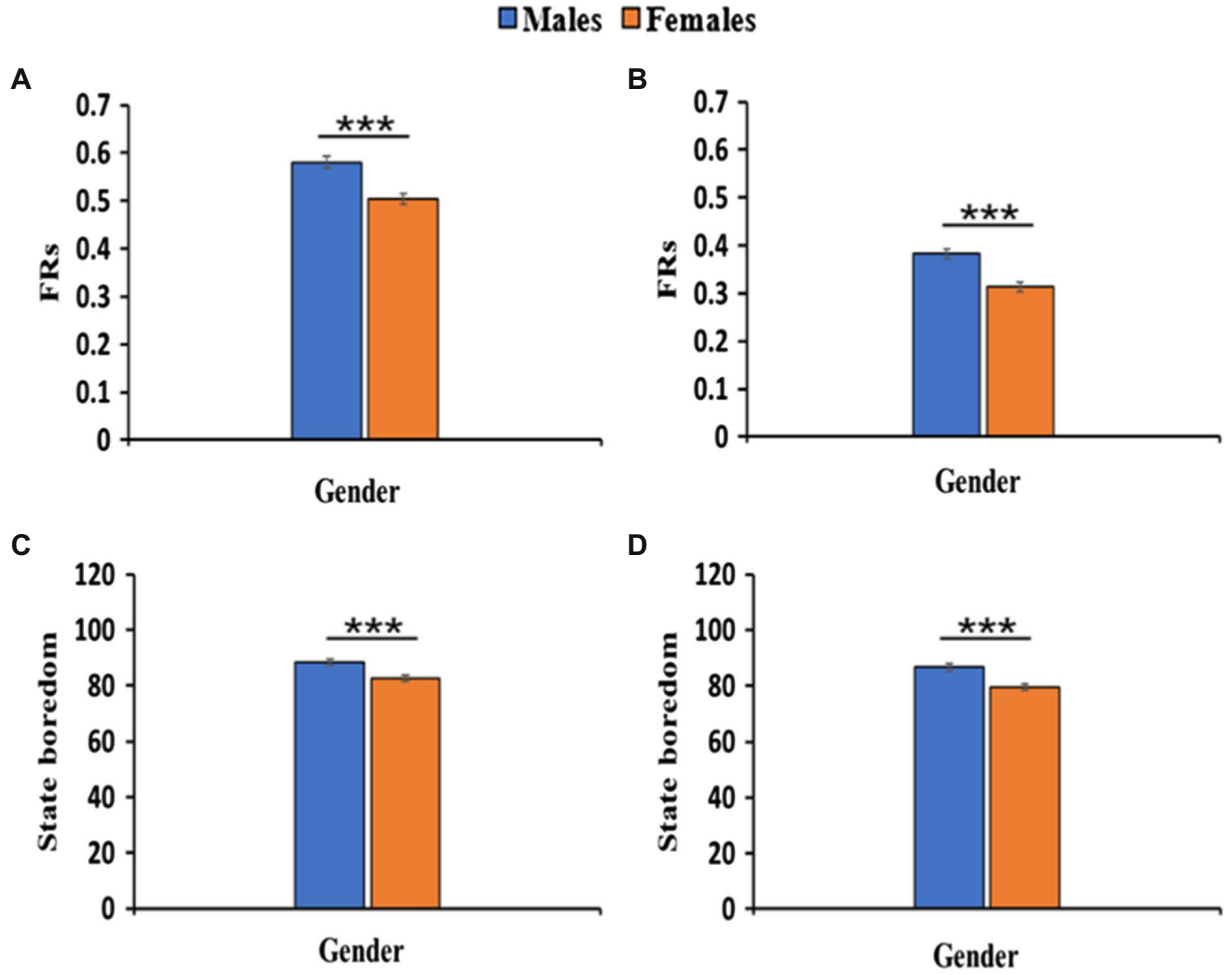
Gender differences for FRs **(A,B)**, scores of state boredom **(C,D)**. ^***^p < 0.001.

Table 2 provides the descriptive statistics of the male and female groups, as well as the differences in FRs, state boredom scores (inattention, time perception, low arousal, high arousal, and disengagement), and trait boredom scores (external stimuli and internal stimuli) between the groups. Consistent with our hypothesis, the male group showed significantly higher FRs than the female group [Experiment 1: *t*(525) = 4.47, *p <* 0.001; Experiment 2: *t*(504) = 3.57, *p <* 0.001, see Table 2, Figures 2A,B], suggesting better performance in female students in the lexicon learning task.

**Table 2:** Descriptive statistics and gender differences for MSBS scores, TBS and FRs of participants.

Experiment 1	Males (*n* = 238)	Females (*n* = 289)	*t*	*p*
FRs	0.58 (0.18)	0.51 (0.17)	4.47	<0.001
TBS	92.02 (12.07)	90.65 (16.02)	1.09	>0.05
Internal stimuli	34.85 (5.53)	34.92 (4.86)	−0.15	>0.05
External stimuli	57.17 (11.45)	55.73 (15.25)	1.20	>0.05
MSBS	88.51 (18.05)	82.68 (20.88)	3.37	0.001
Inattention	19.52 (4.76)	14.80 (5.72)	10.13	<0.001
Time perception	16.42 (6.36)	15.70 (6.33)	1.27	>0.05
Low arousal	18.81 (5.54)	19.28 (6.00)	−0.92	>0.05
High arousal	14.14 (4.97)	13.58 (4.36)	1.33	>0.05
Disengagement	19.86 (5.49)	19.13 (5.48)	1.50	>0.05
**Experiment 2**	**Males (*n* = 228)**	**Females (*n* = 278)**	***t***	***p***
FRs	0.38 (0.22)	0.31 (0.23)	3.57	<0.001
MSBS	86.74 (20.55)	79.53 (21.74)	3.96	<0.001
Inattention	19.63 (4.85)	17.64 (5.47)	4.42	<0.001
Time perception	18.48 (8.49)	18.08 (8.50)	0.53	>0.05
Low arousal	16.43 (5.52)	16.11 (5.42)	0.67	>0.05
High arousal	12.19 (4.86)	12.40 (4.72)	−0.50	>0.05
Disengagement	17.93 (5.46)	16.37 (5.48)	3.27	0.001

MSBS, Multidimensional State Boredom Scale; TBS, Trait Boredom Scale; FRs, Forgetting Rates; Values presented are means (standard deviation).

### Gender and Boredom

**Figure 3:**
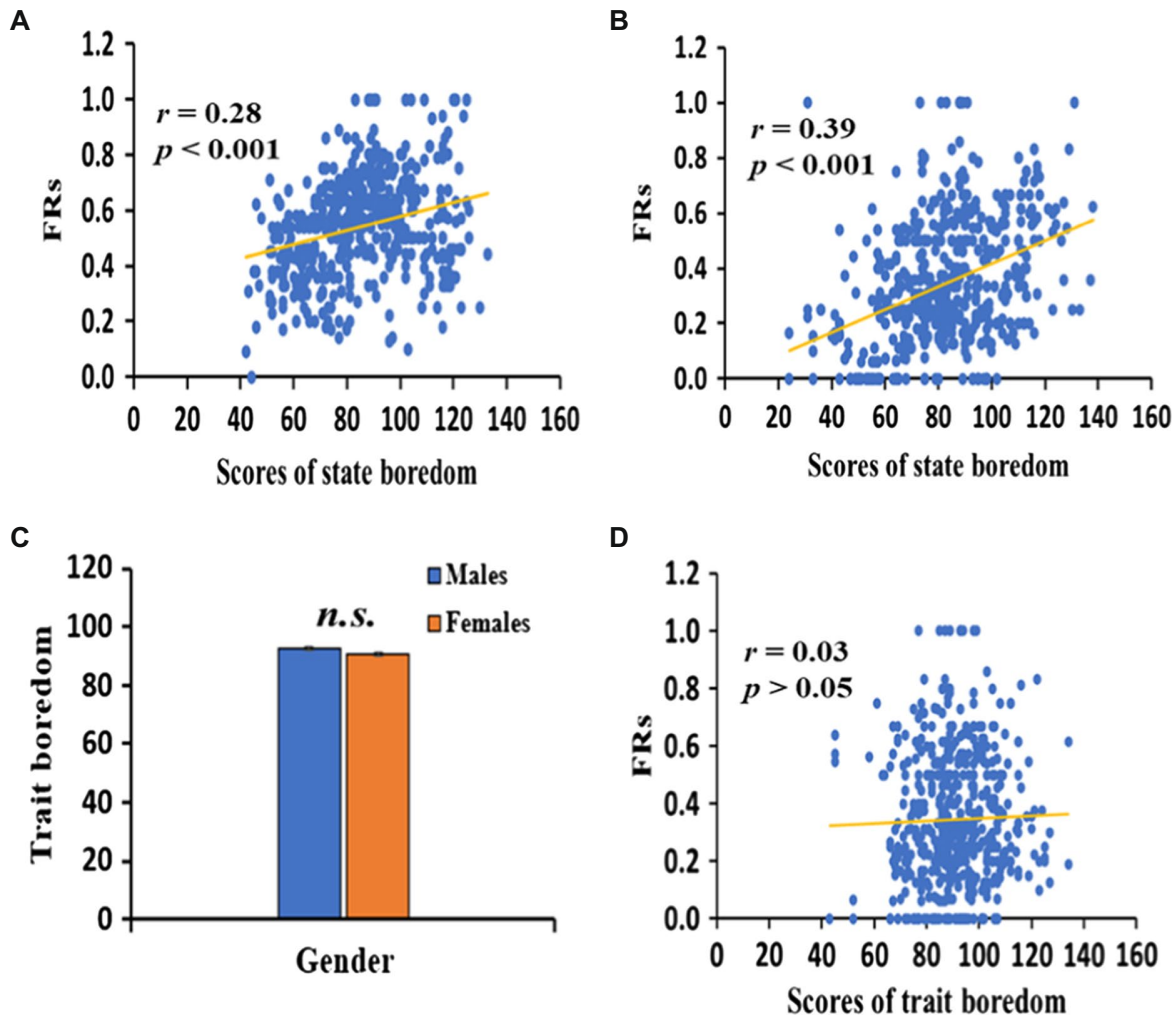
Correlation between FRs and scores of state boredom in Experiment 1 **(A)** and Experiment 2 **(B)**. Gender difference for scores of trait boredom **(C)** and correlation between FRs and scores of trait boredom **(D)** in Experiment 1. n.s. means no significance.

The results also demonstrated distinct gender differences in state boredom. Partly consistent with our hypothesis, the male group showed significantly higher state boredom scores than the female group [Experiment 1: *t*(525) = 3.37, *p* = 0.001; Experiment 2: *t*(504) = 3.96, *p* < 0.001, see Table 2; Figures 2C,D], suggesting a lower level of state boredom in female students before the lexicon learning task. However, inconsistent with our hypothesis, there were no significant differences between the male and female groups in trait boredom scores, [Experiment 1: *t*(525) = 1.09, *p* > 0.05, see Table 2; Figure 3C], suggesting a similar level of trait boredom in female and male students before the lexicon learning task.

### Correlations Between Boredom and Learning Performance

Consistent with our hypothesis, there were significant positive correlations between the state boredom scores and the FRs in both Experiment 1 (*r* = 0.28, *p* < 0.001, see Figure 3A) and Experiment 2 (*r* = 0.39, *p* < 0.001, see Figure 3B), suggesting that high level of state boredom was associated with worse lexicon learning. The stepwise regression was performed to determine what factors in state boredom could be regarded as predictors of FRs. The FRs were used as the dependent variable, and the five dimensions constituting the MSBS were used as the predictor variables. As shown in Table 3, two of the five factors—inattention and low arousal—exerted positive predictive effects on the FRs (Experiment 1: *β* = 0.281, *p* < 0.001; *β* = 0.237, *p* < 0.001; Experiment 2: *β* = 0.225, *p* < 0.001; *β* = 0.210, *p* < 0.001, respectively), the contribution rates reached 16.1 and 4.2%, respectively, in Experiment 1, and 10.0 and 3.6%, respectively in Experiment 2. The results of Experiment 2 replicated that of Experiment 1, showing that inattention and low arousal of state boredom were reliably predictive of novel lexicon learning.

**Table 3:** Stepwise regression analysis of the use of state boredom and FR.

Dependent variable	Independent variable	*R*	*R* ^2^	Δ*R*^2^	*F*	*β*	*B*	*t*
Experiment 1								
FRs	Inattention	0.401	0.161	0.161	100.445	0.281	0.015	6.240^***^
	Low arousal	0.450	0.203	0.042	27.714	0.237	0.013	5.264^***^
Experiment 2								
FRs	Inattention	0.316	0.100	0.100	58.325	0.225	0.010	4.977^***^
	Low arousal	0.369	0.136	0.036	21.721	0.210	0.009	4.661^***^

*** *p < 0.001*.

In contrast to state boredom, no correlations were observed between trait boredom and FRs (*r* = 0.03, *p* > 0.05 in Experiment 1, see Figure 3D), suggesting no significant effects of trait boredom on novel lexicon learning. Taken together, these results suggest that state and trait boredom had different relationships with lexicon learning.

### Gender Effects on Novel Lexicon Learning

We conducted a hierarchical multiple regression analysis to determine the extent to which gender and state and trait boredom could be viewed as predictors of FRs in Experiment 1. Table 4 summarizes the results. Gender was a significant predictor of FRs (*p* < 0.001) and explained about 2.3% of the variance of FRs. When state and trait boredom were included, the model explained about 9.5% of the variance of FRs. State boredom (*p* < 0.001) and gender (*p* < 0.05) were significant predictors of FRs in this model, while trait boredom was not a significant predictor of FRs (*p* > 0.05).

**Table 4:** Hierarchical multiple regression analysis of gender, state and trait boredom as predictors of FR.

Variable	*R*	*R*2	Δ*R*2	*F*	*β*	*B*	*t*
Step 1	0.153	0.023		12.623			
Gender					−0.153	−0.069	−3.553^***^
Step 2	0.309	0.095	0.072	20.745			
Gender					−0.107	−0.049	−2.535^*^
State boredom					0.272	0.003	6.434^***^
Trait boredom					−0.01	0	−0.247

* *p < 0.05;*

*** *p < 0.001*.

*Gender: 1 = male, 2 = female*.

**Figure 4:**
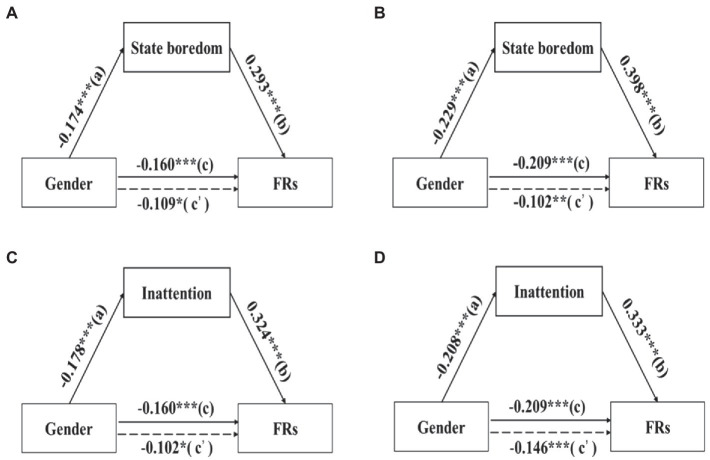
Gender effect on forgetting rates (FRs) was mediated by state boredom in Experiment 1 **(A)** and Experiment 2 **(B)**. Gender effect on forgetting rates (FRs) was mediated by inattention in Experiment 1 **(C)** and Experiment 2 **(D)**. ^*^p < 0.05, ^**^p < 0.01 and ^***^p < 0.001.

The mediation model was further used to explore whether gender, directly or indirectly (through state boredom), affected FRs in both Experiments 1 and 2. The analysis confirmed that gender effect on learning was mediated by state boredom in both experiments (see Figures 4A,B). A bootstrap resampling analysis of the effect size showed that the confidence interval of 95% for gender to influence FRs through state boredom was [−0.03, −0.01] in Experiment 1, and [−0.03, −0.01] in Experiment 2. To explore which dimension of state boredom mediates the relationship between gender and learning outcomes, we also performed a mediation analysis on the dimensions of state boredom. The results indicated that gender effect on novel lexicon learning was mediated *via* inattention in both Experiment 1 and Experiment 2 (confidence intervals were [−0.04, −0.01] and [−0.03, −0.01], respectively, see Figures 4C,D). These results suggested that the inattention dimension of state boredom partially mediated the relationships between gender and novel lexicon learning.

## Discussion

In the present study, two independent experiments with a large sample of over 1,000 college students were conducted to explore the relationships between gender-related differences in boredom and lexicon learning. This study provides converging evidence supporting the advantage of female over male participants in state boredom as well as in novel lexicon learning performance. Moreover, we found that only state boredom, not trait boredom, showed significant effects on lexicon learning, and the differences in state boredom partially explained the differences in lexicon learning outcomes between females and males. These findings suggest that lower-level state boredom in female learners contributes to better language-learning ability in college students.

### Gender Difference in Lexicon Learning

Concerning the novel lexicon learning results, females achieved significantly lower FRs than males. The results were in line with previous findings that females are quite often better in areas involving memorization (Van et al., 2015) and learning strategies (Shukri et al., 2009). The biological viewpoint suggests that gender difference depends on cognitive ability and learning style derived from fundamental physiological differences, such as differences in brain development and higher-level cortical functions (Keefe, 1982). According to the lateralization effect on language, less lateralization for language functions in females could (at least partially) explain why they outperform males in many language skills (Ruigrok et al., 2014). The Gender Role Theory also posits that prevalent gender stereotypes are culturally shared expectations that females and males should learn the appropriate behaviors and attitudes from the sociocultural environment they grow up in (Eagly and Karau, 2002). Consistent with this proposition, females did better than males because females may be more inclined to have the advantage over male learners in learning motivation (Sylvén and Thompson, 2015; Iwaniec, 2019), which subsumes a range of constructs such as positive attitudes and interest (Deci and Ryan, 1985). Consequently, female learners could use a broader range of learning strategies such as cognitive, meta-cognitive, and cognitive refinement strategies than male learners (Gu, 2002). In addition, females’ higher self-regulation (Tseng et al., 2006) and/or more effort investment (Okuniewski, 2014) could contribute to better learning performance. Taken together, the findings that gender impacted novel lexicon learning fit well with the Gender Role Theory (Eagly and Karau, 2002).

### Gender Difference in State Boredom

Our results also indicated that males had higher state boredom than females, which is in line with many previous studies showing that females experienced lower state boredom than males (e.g., Liu et al., 2013; Mehdi, 2021). Males tend to have greater needs for various stimuli, be more active and more risk-seeking, and have greater motivation to seek novel sensations and experiences than females (Mikulas and Vodanovich, 1993; Daschmann et al., 2011; Vodanovich et al., 2011; Burbano et al., 2020). In contrast, females tend to pay more attention to psychological and emotional control and have more strategic competence in coping with experiences of boredom than males (Hogan et al., 2010). However, we did not find significant differences in trait boredom between males and females, suggesting that males and females may have similar structures of trait boredom as stable personality attributes.

### Negative Impacts of State Boredom on Lexicon Learning

We found positive correlations between state boredom scores and the word forgetting rates in both experiments, indicating that higher levels of state boredom are associated with the worse lexicon learning outcome. These results aligned with previous research showing that boredom was related to poor academic achievement (Ahmed et al., 2013; Jaradat, 2015; Suárez-Pellicioni et al., 2016) and more attention deficit (Pekrun et al., 2010; Eastwood et al., 2012; Hunter and Eastwood, 2016; Westgate, 2020). The Control-Value Theory of achievement emotions posits that boredom may result from a lack of control or perceived value in academic tasks (Pekrun, 2006). Therefore, the learners with a high level of boredom may consider the novel lexicon learning as being of little importance or value and perceive control over it as particularly low or high. The aversive state of boredom might trigger their desire to escape the boring situation and at the same time their inability to engage in the learning task.

The findings suggest that state boredom might be characterized in terms of inattention to influence lexicon learning. This helps to explain the relationship between inattention as a dimension of state boredom and learning achievement measures. The interpretation is dependent on the assumption of the CVT, although the adequate measures of control and value appraisals, as the two proximal determinants of achievement emotions when state boredom occurs, are absent in the present research. Further investigations are needed to examine this account: how control and value appraisals contribute to the individual differences in state boredom, respectively. Another possible explanation can be found in the Meaning and Attentional Components model (Westgate and Wilson, 2018), which specifies that the production of boredom was often related to a lack of not only attention but also meaning. Individuals have difficulty concentrating on the current task and are unable to perceive the meaning or importance of a task when state boredom occurs. The fact that state boredom had a significantly negative effect on novel lexicon learning may imply that the learners immersed in high state boredom viewed the issue of novel lexicon learning as less meaningful. Thus, it may contribute to the lack of learning motivation and disengagement from the task at hand, resulting in attention deficit and low arousal in learners. Presumably, repetition of vocabulary memorization made it difficult for the learners to sustain attention and perceive the value of learning. As a result, their academic performance was poor (Malkovsky et al., 2012). Therefore, attention deficit, to some extent, may present novel lexicon learning as meaningless or lack of value, and hence, impacts the performance of lexicon learning. Interestingly, the current study found that state boredom but not trait boredom had significant negative effects on novel lexicon learning. A possible explanation is that different cognitive impairments could be associated with a different type of boredom (Malkovsky et al., 2012). The possible accounts might be different cognitive impairments that could be associated with a particular type of boredom (Malkovsky et al., 2012). However, our current findings cannot ascertain whether this discrepancy was due to the differences between state and trait boredom on neural basis. Future research is needed to elucidate whether state boredom and trait boredom are sufficiently distinct to be treated as separate entities in the brain. In addition, it is unknown whether learning achievements might have influenced learners’ emotions about language learning. Emotions affect learners’ achievement, while experiences of learning outcomes can in turn influence learners’ emotions (Pekrun et al., 2017). This is especially true for the dynamic state of boredom, because determining this fact would require a longitudinal study of the reciprocal causation between boredom and L2 or FLL. This might be an interesting question to expand the present research in the future.

### The Mediating Role of State Boredom

Our results indicated that state boredom partially mediated the interaction between gender and novel lexicon learning. A potential explanation of the mediating role of state boredom reason in lexicon learning may be related to attention, which is influenced by the perceived meaning of a goal or a task. Specifically, weakened attention and mild negative emotions induced by boredom affect the learning process (Pekrun, 2006). When individuals are in a state of boredom, their attentiveness is vulnerable. A lack of attention could drive negative emotions. When attention is not fully engaged, activities would be negatively treated, resulting in poor academic grades or achievement (Hunter and Eastwood, 2016).

Higher-level state boredom has been linked to more inattention and poor achievement based on the boredom mechanism. It is reflected in the findings that males showed significantly higher state boredom and poorer learning effects as compared to females. These findings are in line with the Control-Value Theory of boredom (Pekrun, 2006). The basic structures and causal mechanisms of emotions follow general nomothetic principles. In contrast, the contents, frequency, and intensity of emotions can differ due to different cultures and genders. Regarding gender differences, females’ and males’ emotions should be structurally equivalent as emotions depend on control and value appraisals in both female and male students. To some extent that the perceived control and academic values may differ between female and male students, leading to different emotional experiences. Thus, it is reasonable to expect that state boredom mediates the relationship between gender and novel lexicon learning. Notably, this study has demonstrated that the association between gender and novel lexicon learning is partially mediated by state boredom. Integrating further factors affecting beneficial learning could therefore be important for future studies on this topic. It may be important to examine the relationship between gender and learning achievements by including further individual variables.

### Practical Implications

The findings of this study have important implications for language educators and learners. Boredom is frequently associated with inattention and may be a marker of the emotional status that signals a lack of task value and meaning (Pekrun et al., 2010; Van Tilburg and Igou, 2012). If learning tasks are situationally monotonous and meaningless, learners will feel dissatisfied and disengage from the learning activities (Eastwood et al., 2012). As suggested by the Control-Value Theory, learners’ emotions can be positively influenced by cultivating their ability to perceive and control over academic activities and outcomes, as well as shaping their evaluation of the value of these activities and outcomes (Pekrun, 2006). This shows that students’ boredom experience in the learning context could be potentially attenuated by increasing their sense of control over the task. Students’ boredom may also be attenuated by the enhanced positive feeling of academic values from instruction on task difficulty or importance. Feedback from teachers may play another important role by directing effort to strategies rather than avoidance (Fritea and Fritea, 2013). Cumulative feedback of failures would undermine students’ sense of control and meaning, thus contributing to negative outcomes such as attention deficit. To the extent that this assumption is true, efforts should be made to offer students more opportunities to learn rather than assessing their insufficient attainment.

Findings from this study demonstrate that males had a greater state of boredom and achieved less academic success in novel lexicon learning than females. But this does not mean that males cannot be as effective learners as females. Males tend to adopt cognitive avoidance more than females, who prefer to employ behavioral avoidance to avoid exhausting situations (Mehdi, 2021). Therefore, it may be a good strategy to encourage male students to focus on the utility value of what they are learning to enhance their motivation and minimize boredom during learning (Nett et al., 2010; Tulis and Fulmer, 2013; Coelho et al., 2018). In contrast, female students may be encouraged to use more behavioral avoidance strategies, such as chatting with peers, during the learning task to avoid exhausting situations (Eren and Coskun, 2016). Such gender-specific education strategies may help to reduce students’ boredom in the process of language learning and narrow the gap in language acquisition (Zimmerman, 2014).

### Limitations

The present study had the advantage of enrolling a large sample of over 1,000 healthy and young college students and replicating the main findings in two independent experiments. However, several important limitations should be noted. First, although our findings are in line with the Control-Value Theory which indicates that control and value appraisals play roles in the situation and for the development of boredom, control and value appraisals were not assessed and investigated in this study. Future studies are needed to include the measures of control and value appraisals to further understand their roles in gender differences in boredom and language learning. Second, all participants in this study were young Chinese college students with narrow age ranges; thus it remains unclear whether the present results can be generalized to other age groups such as younger students from primary and secondary schools as well as older learners from the community. Future studies are necessary to replicate the findings in different age populations. Third, since the value appraisals and emotions may differ across countries and cultures (Pekrun, 2006), future research on boredom and learning should be promoted in other countries and cultures. Finally, future studies are needed to use psychophysiological and neuroimaging technologies such as EEG and functional magnetic resonance imaging (fMRI) to determine the neural mechanisms underlying gender differences in boredom and language learning.

## Conclusion

To our knowledge, this is the first study to investigate the contribution of boredom to the gender-related difference in lexicon learning. Findings from two independent experiments with large samples of female and male students consistently demonstrated greater state boredom in male than female participants, which was associated with worse lexicon learning (forgetting more words during the test). Moreover, state boredom but not trait boredom, partially explained the performance difference between male and female participants in the novel lexicon learning task. This study provides new evidence supporting the negative impacts of state boredom on lexicon learning and suggests that better lexicon learning ability in female learners may be partly accounted for by the reduced level of state boredom during learning.

## Data Availability Statement

The datasets used and analyzed in the current study are available from the corresponding author on reasonable request.

## Author Contributions

HR, HW, and YX contributed to the ideas of research and design of research methods. HR, HW, and HS contributed to the collection of data and empirical analysis. XZ, TM, HS, SX, and YH participated in developing a research design and interpreting the analysis. All authors contributed to the article and approved the submitted version.

## Funding

This work was supported in part by the National Natural Science Foundation of China (grant number: 71942003), the school-level Major Research Projects of Shanghai International Studies University (grant numbers: 2021114002, 2021114003, 2017114002, and 2017114005), and the Academic Guidance Project of Shanghai International Studies University (grant number: 41003621). The funders had no role in the study design, data collection and analysis, data interpretation, writing of the manuscript, or the decision to submit the article for publication.

## Conflict of Interest

The authors declare that the research was conducted in the absence of any commercial or financial relationships that could be construed as a potential conflict of interest.

The reviewer QHZ declared a shared affiliation with the author XZ to the handling editor at the time of the review.

## Publisher’s Note

All claims expressed in this article are solely those of the authors and do not necessarily represent those of their affiliated organizations, or those of the publisher, the editors and the reviewers. Any product that may be evaluated in this article, or claim that may be made by its manufacturer, is not guaranteed or endorsed by the publisher.
